# Navigating perinatal mental health integration in maternal and child health services: progress and priorities for research and practices in Pakistan

**DOI:** 10.1192/bji.2024.42

**Published:** 2025-05

**Authors:** Waqas Hameed, Atif Rahman

**Affiliations:** 1 Centre for Global Mental Health, London School of Hygiene & Tropical Medicine, London, UK; 2 Institute of Population Health, University of Liverpool, Liverpool, UK

**Keywords:** Perinatal psychiatry, mental health services, primary care, depressive disorders, psychosocial interventions

## Abstract

We synthesise perinatal mental health (PMH) evidence and provide recommendations for future research and practices in Pakistan. The burden is significantly higher relative to many other countries, with adverse effects on women and children. Few locally developed interventions involving non-specialists have shown promise, but integrating these into maternal and child health services (MCH) at scale remains a challenge. We recommend broadening the scope of PMH research in accordance with the World Health Organization's stepped care model, and advancing the use of implementation science, digital technology and exploring low-cost models. Programmes and policies should prioritise incorporating PMH into MCH services in health planning and budgeting.

Perinatal mental health (PMH) refers to the mental health of women during the pregnancy and extends up to 1 year after childbirth. During the perinatal period, women may experience range of psychiatric disorders, with the most common being depression, anxiety and somatic problems.^1^ In low- and middle-income countries (LMICs), about 19–25% of women during childbirth and 18–19% of women after childbirth experience depression.^2^ Historically overlooked, PMH has garnered significant international attention over the past two to three decades, and is now being incorporated into policies and clinical practices globally.^3^ In this commentary, we synthesised literature on the burden and impact of common perinatal mental disorders (CPMDs) in Pakistan, reviewed existing interventions and policies, and offered recommendations that align with World Health Organization (WHO) guidelines to address identified gaps.

## Our approach

A rapid literature review was conducted in line with the objectives of this commentary. We searched for peer-reviewed articles from Pakistan that address the burden of PMH, associated risk factors, potential consequences and pertinent interventions. Additionally, we specifically examined health policies and laws in Pakistan related to mental health. The search for peer-reviewed articles was carried out on PubMed, focusing primarily on studies from Pakistan. We also used Google, along with WHO and national government websites, to collect international guidelines and information on Pakistan's mental health laws and policies.

## Pakistani context

Pakistan has a population of 241 million,^4^ with one of the highest rates of maternal mortality in the region, and rank riskiest for newborn survival. The prevalence of antepartum (37%) and postpartum (30%) depression^5^ is much higher than the pooled prevalence of LMICs.^2^ However, there is an enormous treatment gap, such that up to 90% of women suffering from perinatal depression remained undiagnosed and untreated^6^ because of the critical shortage of human resource and ill-equipped health systems. To date, PMH services are not integrated into existing maternal and child health (MCH) services within healthcare system. Maternity providers lack capacity for screening and management of CPMDs in the absence of pre-service training. Mental illnesses impose an overall economic cost of approximately US$4.2 billion annually in the country. However, by adopting a gate-keeping role for primary healthcare, potential savings of US$1.6 billion in total costs could be saved.^7^ Unfortunately, only 0.4% of the total healthcare budget is allocated to mental health.

## Determinants of CPMD

**Fig. 1:**
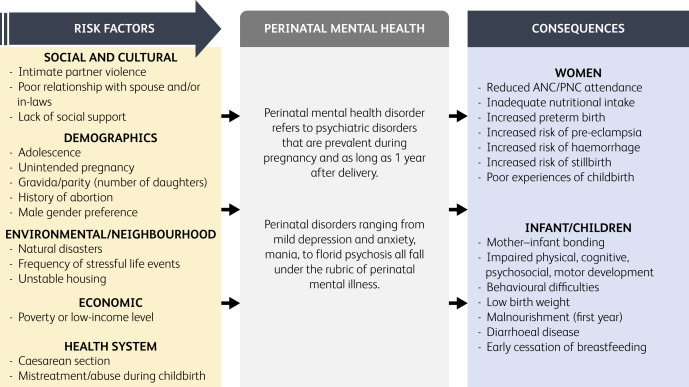
Risk factors and consequences of common perinatal mental disorders. ANC, antenatal care; PNC, postnatal care.

A systematic review of 43 studies identified these commonly reported risk factors,^5^ which are classified according to social determinants framework: social and cultural (intimate partner violence, poor relationship with spouse and/or in-laws, lack of social support), demographics (younger age, unintended pregnancy, number of daughters, history of abortion, male gender preference), environmental/neighbourhood (food insecurity, frequency of stressful life events, unstable housing), economic (poverty or low-income level) and health system (caesarean section, mistreatment of women during childbirth) (Fig. 1).

## Consequences of CPMD

Evidence from LMICs revealed detrimental effects of CPMDs on women's health and well-being of their children, but limited evidence exists from Pakistan. With regard to women, CPMDs (primarily depression) decrease the likelihood of them attending antenatal and postnatal appointments, and increase the risk of obstetric complications such as stillbirth, haemorrhage, premature birth, pre-eclampsia and suicide (Fig. 1). Women with depression are twice as likely to have a premature birth and have 60% higher odds of low-birth weight compared with women without depression. Several longitudinal studies in Pakistan have found that perinatal depression increases the likelihood of behavioural problems and delayed socioemotional development, whereas no association was observed with delayed cognitive and motor development.^8^ Furthermore, children of mothers with depression are more likely to be malnourished in the first year of life,^9^ have higher rates of diarrhoeal disease^9^ and early cessation of exclusive breastfeeding.^10^

## Effective PMH interventions and prevailing gaps

Evidence from LMICs shows that CPMDs, such as anxiety and depression, can be managed in a cost-effective manner by using existing local (non-specialist) professionals to apply evidence-based treatments. Pakistan has also made considerable efforts in this regard.

### Thinking Healthy Programme

A randomised controlled trial demonstrated effectiveness of a psychological intervention (cognitive–behavioural therapy) delivered by community health workers in addressing the issue of perinatal depression.^11^ However, despite promising findings, the model could not be taken to scale because of heavy costs and the low level of feasibility associated with training and supervision of primary healthcare workers. Consequently, the team tested a technological solution to the challenge of training and monitoring, which is found to be as effective as face-to-face training in terms of achieving similar competency levels of service providers, but with significantly less resources.^12^

In another evaluation, the adapted Thinking Healthy Programme (THP) intervention was delivered through peer volunteers in Pakistan and India. The pooled analysis, combining data from both India and Pakistan, showed a significant effect of the THP on reducing symptom severity or achieving remission of perinatal depression at 6 months after childbirth. However, the country-specific analysis for Pakistan revealed a positive effect of THP only at 3 months postpartum. The intervention was deemed less resource-intensive because of the engagement of volunteers, and it was found feasible; however, it encountered issues in relation to fidelity and continued motivation of volunteers in the long-term without financial incentives.

### Happy Mother Healthy Baby

The THP has been adapted to target women with prenatal anxiety, focusing on the mother's health, her relationship with significant others and bonding with her baby. The intervention was found to be effective in reducing common mental disorders like anxiety and depression symptoms,^13^ and was perceived to be culturally acceptable and appropriate.

### Supportive and Respectful Maternity Care

A recent study showed that integration of psychosocial support (adaptation of the WHO Mental Health Gap Action Programme (mhGAP) material) during intrapartum care in facility-based settings can alleviate the prevalence of postpartum anxiety and depression at 42 days,^14^ and improves mother–infant bonding. Although the intervention was co-designed using a participatory consensus-driven process and found to be feasible, the intervention is yet to undergo a larger evaluation to demonstrate its effectiveness in diverse settings.

## Review of mental health laws, policies and programmes

Pakistan's first national mental health policy was developed in 1997 and focused on mental health promotion, integration into primary healthcare and intersectoral collaboration. In 2001, an act was passed that put emphasis on promotive and preventive mental health activities and treatment and management of mental disorders. After health responsibilities were devolved (in 2008) to provincial governments, Sindh, Punjab and Khyber Pakhtunkhwa provinces have passed a mental health act; however, their respective health policies are yet to be formulated. None of said laws specifically talk about PMH. In 2019, the federal and provincial governments have included (but with low priority) integration of mental health into primary healthcare and provision of psychological treatment within the MCH services as essential health interventions in the recently approved Universal Health Coverage (UHC) Benefit Package.

Regarding programmes, the first national mental health programme was initiated in 1987, with the primary aim to integrate mental health services into primary healthcare. In 2000, mental health training was integrated into national teacher training, and school curriculum boards were prompted to include it in their curricula. In 2019, the President′s Programme to Promote Mental Health was launched, emphasising the role of early-life interventions that promote mental health and prevent mental illness. The programme called for a phased implementation of two evidence-based interventions, including THP (through community health workers^11^) for perinatal depression. Despite an initial commitment, little progress has been made to scale-up the interventions, in part because of the COVID-19 pandemic and changed priorities. The programme is receiving renewed attention after promising research on the use of digital technology for scale-up.^15^

## Recommendations for research, programmes and policies

The effective implementation of mental health laws and policies faces significant challenges, including limited financial and human resources, as well as overburdened existing staff. Additionally, gaps in research and implementation evidence hinder the practical integration of proven PMH interventions into the healthcare system. We recommend adopting the WHO's stepped care approach for future PMH research in Pakistan, particularly focusing on promotion of good PMH and preventive interventions for vulnerable women.

We recommend broadening the scope of research on several fronts: (a) PMH research should expand to include anxiety, somatic complaints and mood disorders in addition to depression; (b) intervention models should also cover secondary and tertiary levels of the healthcare system beyond primary healthcare; (c) complementing the cognitive–behavioural therapy and mhGAP materials, there is a need to explore alternative interventions such as problem-solving therapy, behavioural activation, group-based programmes, family-based programmes and mother–baby sessions, to enrich the existing toolkit for practitioners; (d) future studies should consider integrating elements that support both the mother and child's development, recognising the profound impact on mother–child bonding; and (e) a shift toward a preventive focus research, emphasising preventive and promotional strategies aligned with the stepped care model while taking into account social determinants of health and women's psychosocial vulnerabilities.

Researchers must adopt participatory approaches that incorporate human-centred design principles to ensure that interventions align with the sociocultural context and address the needs, priorities and preferences of women and children. There is a need to employ methods of implementation research to gain deeper insights and a clearer understanding of the potential challenges that may arise when delivering these interventions in real-world settings. This should be complemented by rigorous economic evaluations to provide valuable insights into the financial aspects of scaling up effective interventions. Furthermore, the potential of digital technology should be explored to facilitate training, supervision and delivery of PMH interventions. Developing human resources, such as utilising peers and other non-specialist providers, can help expand intervention access. Investigating public–private partnership models may help overcome financial barriers to implementation.

With respect to policies and programmes, the President's Programme to Promote Mental Health should establish a clear strategic plan with defined short- and long-term milestones, prioritising the integration of PMH into MCH services as previously promised. Moreover, federal and provincial governments are urged to formulate mental health policies that prioritise PMH, allocating adequate budgets for its implementation and ensuring that PMH is included in the priority list of the UHC Benefit Package. These combined efforts aim to significantly enhance the scope and effectiveness of PMH interventions and policies, aligning them more closely with the needs and challenges faced by women and children during the perinatal period.
